# Parietal and occipital leukoaraiosis due to cerebral ischaemic lesions decrease the driving safety performance of healthy older adults

**DOI:** 10.1038/s41598-022-25899-4

**Published:** 2022-12-12

**Authors:** Hikaru Oba, Kaechang Park, Fumio Yamashita, Shinichi Sato

**Affiliations:** 1 Graduate School of Health Sciences, Hirosaki University, 66-1, Hon-Cho, Hirosaki, Aomori 036-8564 Japan; 2 Traffic Medicine Laboratory, Research Organization for Regional Alliance, Kochi University of Technology, 185 Miyanokuchi Tosayamada-Cho, Kami, Kochi 782-0003 Japan; 3 Division of Ultrahigh Field MRI, Institute for Biomedical Sciences, Iwate Medical University, 1-1-1 Idaidori, Yahaba-Cho, Shiwa-Gun, Iwate, 028-3694 Japan; 4 Graduate School of Human Sciences, Osaka University, 1-2, Yamadaoka, Suita, Osaka 565-0871 Japan

**Keywords:** Neuroscience, Health care, Engineering

## Abstract

Leukoaraiosis, a common ischaemic lesion diagnosed using magnetic resonance imaging (MRI), can influence driving safety performance (DSP). Most older drivers with leukoaraiosis are unaware of their affliction. Japan is a super-aged country, where preventing accidents caused by older drivers is an urgent national issue. We investigated the subcortical and periventricular leukoaraiosis regions that were most involved in DSP decline. The driving skills of 101 drivers (49 men, 52 women; mean age, 77.88 ± 3.77 years) without dementia were assessed by official driving instructors, using actual vehicles on a closed-circuit course. Parietal and occipital (but not frontal or temporal) leukoaraiosis volumes were significantly correlated with decreased DSP scores regardless of age, especially when turning right at intersections, which needs more attention than turning left because left-side driving is legally enforced in Japan. Occipital leukoaraiosis was also involved via a decline in dynamic visual cognitive function. MRI-based assessment of leukoaraiosis volume and localisation may enable the identification of older drivers prone to DSP deterioration. Risk factors for leukoaraiosis include smoking and lifestyle-related diseases such as hypertension. Thus, brain healthcare in patients with MRI-diagnosed leukoaraiosis may be particularly useful for the risk management of traffic accidents caused by the elderly in Japan.

## Introduction

The World Health Organization and the United Nations define ageing rates (the percentage of the population aged ≥ 65 years in relation to the total population) above 7%, 14%, and 21% as markers of an ‘ageing society’, ‘aged society’, and ‘super-aged society’, respectively^1^. In the super-aged society of Japan, with an ageing rate of 28.8% in 2020^2^, measures to prevent traffic accidents caused by older drivers have become a national challenge to overcome urgently^3^. However, the decline in driving ability varies greatly among older drivers, which is a major barrier to introducing measures targeting older people unable to drive safely^4–6^. It is well known that traffic accidents involving younger and older drivers differ in characteristics^7^. Whereas young people often drive aggressively at excessive speed, older people are involved in traffic accidents at crossroads regardless of driving speed^7–9^. In recent years, the number of severe accidents, particularly in older drivers and seldom seen in young drivers, is increasing, including accidents involving incorrect entry into highways and incorrect operation of the accelerator and brake pedals at the time of starting or parking^3^. Therefore, specific tests or examinations are required for the early detection of poor driving-related abilities such as the decline in executive function required to quickly respond to dangerous situations.

We advocate for the hypothesis that leukoaraiosis (LA) can contribute to dangerous driving performance in older drivers. LA is regarded as an ischaemic lesion due to arteriosclerosis of small blood vessels in the cerebral white matter^10,11^. In general, LA increases in frequency with age and is found in at least 40% of apparently healthy adults over the age of 50 years, with some reports estimating this frequency as high as 95% in the elderly^11,12^. LA is classified into four grades according to its extent within the cerebral white matter. Grade 4 LA has been reported to be associated with recurrent cerebral stroke and vascular dementia^10^. Recent functional magnetic resonance imaging (fMRI) studies have reported functional disturbances associated with LA^13,14^. Such dysfunction of neuronal networks may be caused by damaged vessels in patients with LA providing insufficient blood supply to the white matter, including neuronal fibres. Through interference with neuronal communication involving white matter, LA is regarded to affect safe driving abilities, such as planning, prioritising, risk assessment, processing speed, and attention, which depend on the rapid and accurate exchange of information^15–17^. Driving safety performance (DSP) is naturally based on these abilities, suggesting a relationship to LA^18^. DSP can be evaluated by publicly certified driving instructors using actual vehicles on the road. In addition, decreases in dynamic visual cognition (DVC) scores, which can be measured using a computer-based driving aptitude test (Dynamic vigilance checker), are significantly correlated with increases in LA grade^19,20^. Our previous study of 3930 healthy middle-aged drivers showed a significant association between LA grade and the incidence of crossroads accidents in the past 10 years^21^. Another study examined the relationship between DSP and LA in older drivers in the presence of distraction tasks. Participants had to drive vehicles on a closed-circuit course while adding numbers heard from the car stereo at two-second intervals while driving^22^. The authors reported that healthy older drivers with higher LA grades produced more driving-related errors, such as ignoring stop signs, failing to confirm a safe right-of-way by checking both sides at intersections, and steering disturbances, compared to those without LA. In another driving test conducted on a closed-circuit course without distracting tasks, LA grades were statistically correlated with lower DSP scores when brain atrophy (BA) and LA grades were used as explanatory variables^23^. This indicated that BA may have a similar effect to distractors when LA affects DSP. These results clearly demonstrated the effect of LA on DSP. Thus, a lower LA grade may be a human factor (a technical term of psychology, engineering, and transportation science) that reduces human error, increases productivity, and enhances safety and comfort, with a specific focus on the interaction between humans and automobiles.

In all studies thus far, LA was qualitatively classified based on MRI imaging by medical specialists; LA volume and localisation have never been evaluated in relation to DSP assessment^21–23^. A method to automatically measure LA volumes was recently developed^24^ and has been used to elucidate in detail the correlations with various DSP tasks like changing lanes while driving straight, driving along a large curve with poor visibility, turning right and left at intersections, and stopping at signs. The present study investigated the relationships between DSP and LA volume for the first time. Furthermore, age, DVC score, brain structure data related to BA and LA including lesion localisation, and the abovementioned DSPs using actual vehicles at various locations on a closed-circuit track were also investigated. The results may improve our understanding of brain factors associated with DSPs in humans and identify healthy older drivers at high risk of dangerous driving using MRI data.

## Materials and methods

### Participants

The study population comprised 101 participants (49 men, 52 women; mean age 77.88 ± 3.77 years) who were older people living in the Chugei area of Kochi Prefecture in Japan recruited through local newspapers and television. Each participant underwent an MRI examination at Tano Hospital, a medical centre in the Chugei area. A dementia specialist (K.P.) interviewed all participants with their families, examined the participants, and ruled out dementia based on MRI findings and neuropsychological tests including the Conversational Assessment of Neurocognitive Dysfunction, newly developed for dementia diagnosis based on daily conversations^25^. We collected the medical histories of participants and confirmed that none had cerebrovascular disorders, brain tumours, or cardiovascular disease. The DSP of the enrolled participants was also evaluated in actual vehicles on roads at the Aki Driving School located in the Chugei area of Kochi. For enrolment, participants had to drive > 2 times and > 5 km per week. Professional drivers were excluded from the study.

### Ethics statement

The present study was conducted in accordance with the ‘Ethics Guideline for Medical and Health Research Involving Human Subjects’ based on the Declaration of Helsinki. All participants signed a formal agreement outlining that the experimental data will only be used for scientific purposes and that their anonymity would be protected. Written informed consent was obtained from all participants. The present study was approved by the Institutional Review Board of the Kochi University of Technology (application number: C4-3).

### Measurement of LA volume

A 1.5-Tesla MRI system (ECHELON Vega; Hitachi Medical Corporation, Tokyo, Japan) was used for the MRI-based LA diagnosis. As described previously^24^, the protocol included T2-weighted spin-echo (repetition time/echo time [TR/TE] = 5800/96 ms), T1-weighted spin-echo (TR/TE = 520/14 ms), and fluid-attenuated inversion recovery (FLAIR; TR/TE = 8500/96 ms; inversion time = 2100 ms) imaging. Images were obtained from 27 transaxial slices per scan. The slice thickness was 5 mm with no interslice gaps. In this study, LA areas were automatically segmented, and the LA volume was quantified as previously reported^26^. In short, FLAIR images were corrected for intensity inhomogeneity using unified segmentation module implemented in Statistical Parametric Mapping 12^27^. The intensity inhomogeneity-corrected image (IICI) was then anatomically normalised into the template space using advanced normalisation tools^28^, and a region-of-interest delineating the middle cerebellar peduncle was applied to the anatomically normalised IICI to estimate the intensity distribution of normal white matter in each participant. The voxel values of the IICI in the native space were then normalised to have a mean value of 1000 and a standard deviation of 100 in the middle cerebellar peduncle. The intensity-normalised IICI was thresholded using a cut-off of 3.5 standard deviations to segment LA, with search regions limited to the LA mask. LA volume was calculated by multiplying voxel size and slice thickness. Finally, the measured LA was automatically coloured red to be validated by one of the authors (K.P.), a neurosurgeon trained to confirm the presence and location of LA. For detailed regional analysis, the segmented LA was parcellated into four cerebral lobes and four lateral ventricles as previously reported^29^.

### Measurement and definition of brain atrophy

T1-weighted MR images were obtained using the 1.5-Tesla ECHELON Vega system with three-dimensional gradient-echo inversion recovery sequences. The following scanning parameters were used: TR, 9.2 ms; TE, 4.0 ms; inversion time, 1,000 ms; flip angle, 8°; field of view, 240 mm; matrix size, 0.9375 × 0.9375 mm; slice thickness, 1.2 mm; and number of excitations, 1. Each image was visually assessed for brain diseases and anomalies. Imaging interference with head motion and artefacts were excluded for analyses because of the affected volumetric measurement. The images were processed and analysed using the Voxel-based Morphometry 8 toolbox (http://dbm.neuro.uni-jena.de/vbm/) and other modules implemented in Statistical Parametric Mapping 8 (https://www.fil.ion.ucl.ac.uk/spm/) to determine regional cerebral volumes^26,30^. Images were segmented into grey matter, white matter, and cerebrospinal fluid space using the maximum posteriori approach as previously described^23^. The segmented grey matter and white matter images were then used to estimate the morphological correspondence between the template image and the participant’s brain using a high-dimensional non-linear warping algorithm^26^. The estimated non-linear warp was inversely applied to an atlas defined in the template space to anatomically parcellate the target brain. The neuromorphometric atlas incorporated in Statistical Parametric Mapping 12 was used for parcellation, with a modification for white matter lesions, which appeared as incorrect grey matter segments around the lateral ventricles. The volume of each anatomical region was calculated as the sum of the corresponding tissue densities in the voxels belonging to each region. Intracranial volume (ICV) was defined as the sum of the total brain volume (TBV) and cerebrospinal fluid volume (CFV). BA was defined as the ratio of CFV to ICV, i.e., (ICV − TBV)/ICV = 1 − TBV/ICV.

### Evaluation of driving safety performances

**Figure 1:**
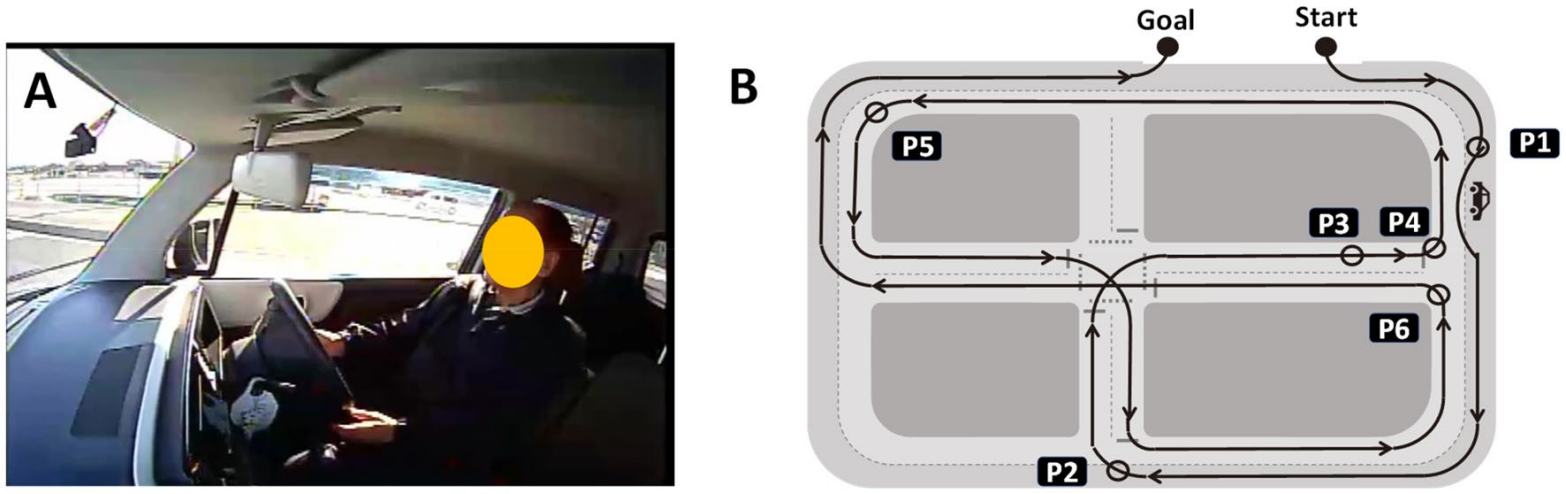
Driving a vehicle on a closed-circuit course. (**a**) Driver’s appearance as seen from the passenger seat. (**b**) Map of the closed-circuit course with six rating points. P1, changing lanes while driving straight; P2, turning right at an intersection; P3, straight course; P4, turning left at an intersection with a stop sign; P5, large curve with poor visibility; P6, turning left at an intersection. The corresponding author owns the copyright of the photograph.

Experiments involving the driving of actual vehicles were performed on a closed-circuit course (Fig. 1a), officially designated by the National Police Agency for renewal of driving licences for elderly people^31^. The course was located in the Aki Driving School in Chugei, Kochi Prefecture, Japan (Fig. 1a). In this test, six locations on the driving course were selected for rating DSPs as previously described^23^. The tasks included changing lanes while driving straight (location P1 in Fig. 1b), turning right at an intersection (P2), driving a straight course (P3), turning left at an intersection with a stop sign (P4), driving a large curve with poor visibility (P5), and turning left at an intersection (P6). It should be noted that, in Japan, vehicles are legally driven on the left side of the road. No further driving events were included in this test.

In this test, a four-wheeled 1400-cc vehicle (Comfort; Toyota, Aichi, Japan) was used. The speed of a vehicle on the closed-circuit track is typically 20–50 km/h, and it takes approximately 20 min to complete the course. Before the test, a qualified driving instructor drove the course, demonstrating good driving performance with the participant sitting next to the instructor. The participant then drove with the evaluating instructor sitting in the passenger seat. The instructor rated the driving skills of each participant using a previously described method^23,32^. DSP scores at the six locations (P1–P6) were then calculated by assessing some of the six categories depending on the location: ‘searching (safety recognition and behaviour with head movement)’, ‘speeding (choice of vehicle speed)’, ‘signalling (the timely and appropriate usage of the indicator)’, ‘stability (acceleration and braking without knocking and completely pulling up in front of the stop line)’, ‘positioning (vehicle movement along the curvature radius at intersections without large or small turns)’, and ‘steering (smooth handling with appropriate starting and ending)’. Each category was evaluated using a three-point scale: 1 (poor performance), 2 (normal performance), and 3 (excellent performance). P1 was evaluated for signalling, searching, and steering; P2 for searching, speeding, signalling, and positioning; P3 for searching and speeding; P4 for searching and stability; P5 for searching and speeding; and P6 for searching, speeding, signalling, and positioning. Higher DSP scores indicated stronger adherence to safe driving standards mandated by the Road Traffic Act in driving guidance for older drivers when renewing their licenses.

### Dynamic visual cognition test

The DVC test (Micromate Okayama Co., Ltd.) is a driving aptitude test that has been used as an auxiliary evaluation for the renewal of driving licences at Aki Driving School^33^. The device used in this test performs two functions: a motion tracking function that follows dynamic indicators mainly using smooth pursuit movements and a function to detect sudden motion and jumping indicators mainly using saccadic eye movements (Supplementary Fig. 1a,b with movie)^19,20^. The participants were required to press a button with their dominant hand when discovering the target signal, a closed octagon. This was considered a ‘positive response’. If the button was not pressed when the target signal was presented or the button was pressed when the noise target (an open octagon) was presented, a ‘false response’ was recorded. The moving body recognition rate (DMD ratio), as a variable in either tracking or sudden motion tasks, was calculated by dividing the sum of positive responses to signals and false responses to noise by the total number of signals and noises displayed:

$$\mathrm{DMD \; ratio }= \frac{\mathrm{No}.\; \mathrm{ of \; positive \; responses }\left(\mathrm{signal}\right)+\mathrm{No}. \; \mathrm{ of \; false \; responses }\left(\mathrm{noise}\right)}{\mathrm{Total \; no}. \; \mathrm{ of\; responses }\left(\mathrm{signal \; and \; noise}\right)}.$$

**Figure 2:**
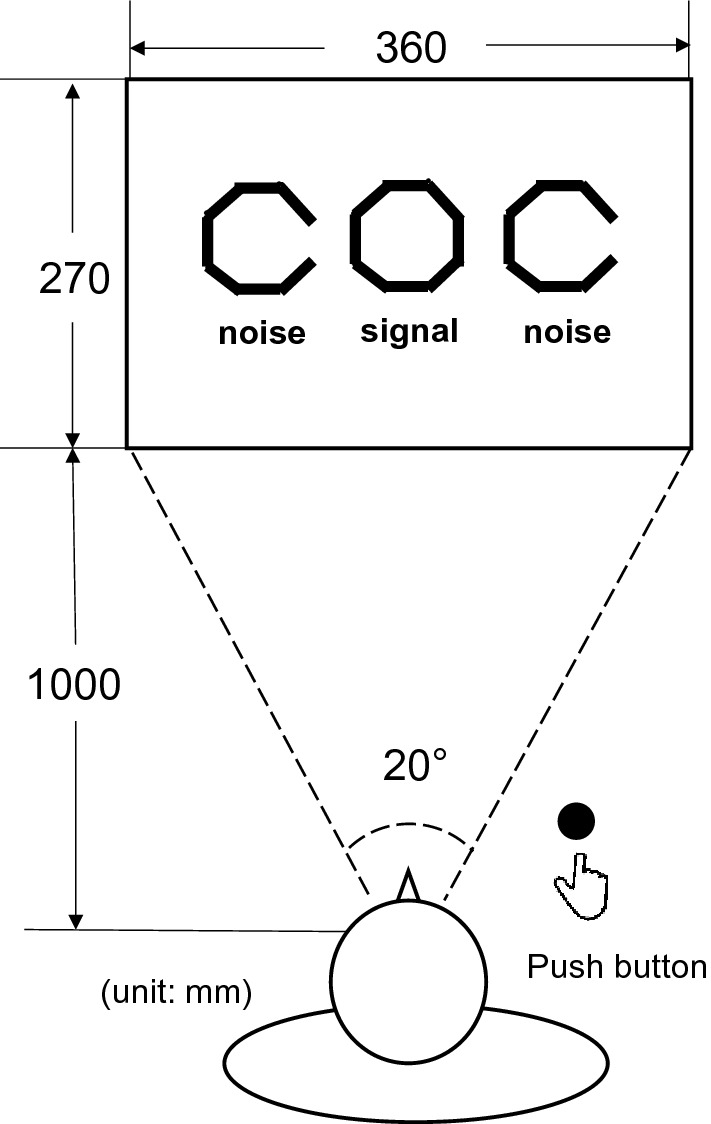
Dynamic visual cognition test.

The participant sat in front of a computer screen (360 × 270 mm). The distance between the participant’s face and the screen was 1000 mm, with a viewing angle of 20° (Fig. 2). Participants wore glasses if necessary. First, they practised the tracking and sudden motion tasks for approximately 20 s. The time required for the entire test was 7–8 min.

### Statistical analysis

BA values and LA volume values of the total white matter, frontal, temporal, parietal, and occipital lobes, as well as periventricular areas, were used as brain structure data. All periventricular areas were integrated into one variable because of multicollinearity. Pearson’s correlation coefficient and regression analyses were conducted to examine the relationships between brain structure data represented by LA volumes and driving performances. In the regression analysis, we examined how DVC and brain structure were associated with DSP after adjusting for age. An additional model in which DVC was set as the dependent variable was used to explore how the brain structure affects DVC scores. Path analysis was performed to confirm the model fit of the relationships among brain structure, DVC, and driving performance. The model fit was confirmed using chi-squared statistics, the goodness of fit index (GFI), the adjusted goodness of fit index (AGFI), root mean square error of approximation (RMSEA), and Akaike’s information criterion (AIC). Non-significant chi-squared statistics, higher GFI and AGFI (> 0.90), lower RMSEA (< 0.05), and lower AIC values indicated a good model fit. The significance level was set at *p* < 0.05; all statistical analyses were performed using R version 4.0.3, and path analysis was conducted using the ‘lavaan’ package^34^.

## Results

The descriptive statistics of the brain structure data and DSP scores are shown in Tables 1 and 2. Pearson’s correlation analysis showed significant correlation between the sudden motion task of the DVC and DSP scores while turning right at an intersection (r = 0.30, *p* < 0.01) as well as between parietal LA volume and DSP scores for a temporary stop at an intersection (r = − 0.20, *p* < 0.05). The sudden motion task of the DVC was also correlated with occipital LA volume (r = − 0.25, *p* < 0.05). Table 3 shows the results of the univariate correlation analysis for all variables.

**Table 1:** Descriptive characteristics of brain structures (*n* = 101).

	Mean	Standard deviation	Min–Max
Brain atrophy	0.23	0.02	0.16–0.29
**LA volume**			
Total	14.7	15.8	0.46–102.5
Frontal	0.62	1.77	0–14.83
Temporal	0.02	0.04	0–0.25
Parietal	0.14	0.39	0–2.26
Occipital	0.001	0.004	0–0.04
Periventricular	14.6	14.5	0.5–89.5

*LA* leukoaraiosis.

**Table 2:** Descriptive characteristics of DVC tests and DSPs using actual vehicles.

	Mean	Standard deviation	Min–Max
DVC, tracking motion task	89.6	5.6	72–98
DVC, sudden motion task	92.9	5.4	76–99
**DSP score**			
Changing lanes while driving straight (P1)	5.8	1.5	3–9
Turning right at an intersection (P2)	11.1	1.0	7–12
Straight course (P3)	2.5	1.2	2–6
Turning left at an intersection with a stop sign (P4)	4.9	1.4	2–6
Large curve with poor visibility (P5)	4.5	1.1	2–6
Turning left at an intersection (P6)	9.6	1.6	4–12
Total DSP score	38.3	4.8	26–51

*DVC* dynamic visual cognition, *DSP* driving safety performance.

**Table 3:** Univariate correlation analysis of all variables.

		1	2	3	4	5	6	7	8	9	10	11	12	13	14	15	16
1	Age																
	DSPs																
2	Changing lanes while driving straight (P1)	− 0.09															
3	Turning right at an intersection (P2)	− 0.16	0.31**														
4	Straight course (P3)	− 0.13	0.22*	0.17													
5	Turning left at an intersection with a stop sign (P4)	− 0.02	0.25*	0.14	0.04												
6	Large curve with poor visibility (P5)	− 0.07	0.13	0.37***	0.34***	0.21*											
7	Turning left at an intersection (P6)	0.06	0.20*	0.49***	0.22*	0.36***	0.48***										
8	Total DSP score	− 0.09	0.59***	0.63***	0.51***	0.57***	0.66***	0.77***									
9	DVC, tracking motion task	− 0.21*	0.13	0.03	0.05	0.18	− 0.07	0.01	0.10								
10	DVC, sudden motion task	− 0.18	0.11	0.30**	0.05	− 0.02	− 0.02	0.03	0.11	0.50***							
11	Brain atrophy	0.35***	0.15	− 0.06	0.03	− 0.06	− 0.1	0.07	0.02	− 0.18	− 0.27*						
	Leukoaraiosis volume																
12	Frontal LA	0.19	− 0.12	− 0.09	− 0.07	− 0.17	0.01	− 0.1	− 0.15	− 0.20*	− 0.10	− 0.01					
13	Temporal LA	0.06	0.02	− 0.03	− 0.02	− 0.18	0.03	− 0.08	− 0.08	− 0.19	− 0.13	− 0.17	0.73***				
14	Parietal LA	0.20	− 0.11	− 0.16	− 0.12	− 0.20*	− 0.05	− 0.12	− 0.20*	− 0.17	− 0.11	− 0.04	0.86***	0.69***			
15	Occipital LA	0.12	− 0.05	− 0.06	− 0.04	− 0.01	− 0.07	0.02	0.05	− 0.09	− 0.25*	− 0.05	0.19	0.31**	0.29**		
16	Periventricular LA	0.33**	− 0.05	− 0.07	− 0.04	− 0.16	0.07	0.02	− 0.06	− 0.21*	− 0.16	0.16	0.78***	0.54***	0.79***	0.17	
17	Total LA volume	0.33**	− 0.06	− 0.08	− 0.04	− 0.16	0.06	0	− 0.08	− 0.22*	− 0.16	0.15	0.82***	0.57***	0.83***	0.18	1.00***

Significant values are in bold.

*DSP* driving safety performance, *DVC* dynamic visual cognition, *LA* leukoaraiosis; *p < 0.05, **p < 0.01, ***p < 0.001.

Multiple regression analysis showed that both parietal LA and the sudden motion task of the DVC were significantly associated with DSP scores while turning right at an intersection (β = − 0.46, *p* < 0.05 for parietal LA; β = 0.41, *p* < 0.001 for DVC score in the sudden motion task). Although temporal LA and BA were significantly associated with DSPs in the task consisting of changing direction, i.e., at the point of P1, the statistical parameters indicated a poor fit (F [9, 91] = 1.54, *p* = n.s.) (Table 4).

**Table 4:** Relationships of DSP scores with leukoaraiosis and brain atrophy.

	P1	P2	P3	P4	P5	P6
Age	− 0.12	− 0.16	− 0.15	0.06	− 0.08	0.03
DVC, tracking motion task	0.10	− 0.18	0.02	0.22	− 0.08	− 0.01
DVC, sudden motion task	0.13	0.41***	0.05	− 0.17	− 0.02	0.08
Brain atrophy	0.27*	0.04	0.08	− 0.12	− 0.15	0.03
**Leukoaraiosis volume**						
Frontal	− 0.31	0.02	− 0.02	0.12	0.02	− 0.12
Temporal	0.36*	0.14	0.12	− 0.12	0.06	0.06
Parietal	− 0.16	− 0.46*	− 0.31	− 0.24	− 0.39	− 0.34
Occipital	− 0.01	0.09	0.02	0.03	− 0.05	0.08
Periventricular	0.17	0.26	0.20	0.01	0.37	0.33
F (df1, df2)	1.54 (9, 91)	2.28 (9, 91)*	0.53 (9, 91)	1.09 (9, 91)	0.84 (9, 91)	0.67 (9, 91)
R^2^	0.13	0.18	0.05	0.10	0.08	0.06
Adj R^2^	0.05	0.10	− 0.04	0.01	− 0.01	− 0.03

Significant values are in bold.

*DSP* driving safety performance, *DVC* dynamic visual cognition, *P1* changing lanes while driving straight, *P2* turning right at an intersection, *P3* straight course, *P4* turning left at an intersection with a stop sign, *P5* large curve with poor visibility, *P6* turning left at an intersection; *p < 0.05, ***p < 0.001.

**Figure 3:**
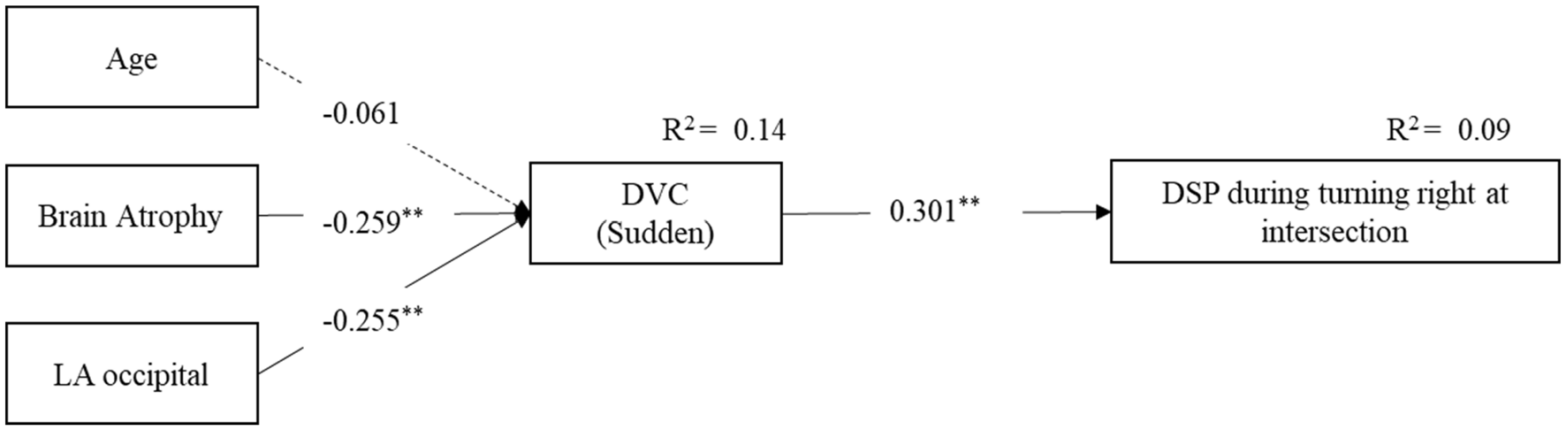
Final model. The dotted arrow indicates a non-significant path. Chi-squared (df = 3) = 1.9, *p* = 0.602, GFI = 0.993, AGFI = 0.964, RMSEA = 0.000, AIC = 1411.3. *DSP* driving safety performance, *DVC* dynamic visual cognition, *LA* leukoaraiosis, *GFI* goodness of fit index, *AGFI* adjusted goodness of fit index, *RMSEA* root mean square error of approximation, *AIC* Akaike’s information criterion.

Since the sudden motion task of the DVC was strongly associated with driving performance, we further analysed the relationships between brain structure data and DVC scores (Table 5). The results showed that occipital LA and BA were significantly associated with DVC scores for the sudden motion task (β = − 0.23, *p* < 0.05 for occipital LA; β = − 0.27, *p* < 0.05 for BA). Path analysis was then used to confirm the DSP model fit. Although age was a non-significant variable affecting DVC scores, it was treated as an explanatory variable to better describe DVC effects. The results indicated excellent model fitness (chi-squared [df = 3] = 1.9, *p* = 0.602, GFI = 0.993, AGFI = 0.964, RMSEA = 0.000, AIC = 1411.3; Fig. 3).

**Table 5:** Relationship between leukoaraiosis volume and DVC scores.

	DVC, tracking motion task	DVC, sudden motion task
Age	− 0.12	− 0.04
Brain atrophy	− 0.16	− 0.27*
**Leukoaraiosis**		
Frontal LA	− 0.10	0.06
Temporal LA	− 0.18	− 0.15
Parietal LA	0.12	0.10
Occipital LA	− 0.04	− 0.23*
Periventricular LA	− 0.06	− 0.12
F (df1, df2)	1.56 (7, 93)	2.49 (7, 93)*
R^2^	0.11	0.16
Adj R^2^	0.04	0.10

*LA* leukoaraiosis, *DVC* dynamic visual cognition; *p < 0.05.

## Discussion

As we have previously reported based on questionnaire surveys and vehicle driving assessments, we consider minor LA as a human factor in the brain affecting DSP^21–23^. In the present study, we investigated in which cerebral lobes signs of LA were detected and how these lobes were involved in the decline of DSP. Our results showed a significant correlation of parietal LA with DSP scores. We further found a correlation between occipital LA and DVC. These results are plausible because the parietal and occipital lobes are involved in spatial and visual cognition, respectively^35^. However, the lack of correlation with frontal and temporal LA was unexpected because the frontal and temporal lobes carry out executive function and object recognition, respectively, which are essential for DSP^35^. In a statistical model, however, parietal and occipital LA were significantly associated with DSP deterioration only when periventricular LA and BA were used as explanatory variables. Since periventricular LA and BA were definitively located in the frontal and temporal lobes, there may not be any significant association but rather a subtle correlation between the frontal and temporal function and DSP. Furthermore, although age was not a significant factor, it is important for understanding the relationships between LA and human factors in the brain and establishing preventive measures against traffic accidents caused by older drivers. Ageing is unavoidable, and although LA volume increases with age, this increase depends to some extent on healthcare and medical measures^10–12^. LA is greatly affected by lifestyle factors such as smoking^36,37^ and lifestyle-related diseases such as metabolic syndrome^38,39^, hypertension, and diabetes mellitus^12,24^. For instance, people with hypertension are up to 14 times more likely to develop LA than those without hypertension^40^. Comprehensive antihypertensive measures include blood pressure control with or without drug treatment, reduced salt intake, regular physical exercise, dietary control, and smoking cessation^12,24^. On the other hand, conventional measures for the prevention of traffic accidents include heightened awareness of traffic rules, punitive approaches to drunk driving, improved road infrastructure, and enhanced vehicle safety^7,8^. This does not include approaches targeting LA, such as lifestyle changes and medical treatments. In addition to conventional approaches, measures alleviating LA might lead to the prevention of traffic accidents regardless of age. However, the decline in DSP as a cause of traffic accidents has not been reported yet. An analysis of the Japanese traffic accident database revealed that drivers frequently committing traffic violations tend to be more prone to be the cause of traffic accidents^9^. Traffic violations are expected to occur if drivers do not follow the Road Traffic Act and check items, which are the basis for the DSP evaluation conducted in this study^7,8^. Therefore, MRI examinations may facilitate the identification of potentially dangerous drivers regardless of age based on LA volume and localisation in the parietal and occipital lobes. This may help to prevent traffic accidents caused by a decline in DSP based on better LA management. Whereas MRI examination remains too expensive for preventive medicine, healthcare check-ups using an MRI procedure called Brain Doc have been uniquely developed and their use prevails in Japan^38,39^. By applying the LA volume algorithm used in this study, it is possible to accurately measure LA volume even with the 1.5-Tesla MRI commonly used in Brain Doc. In this study, we proposed an innovative MRI-based measure to prevent traffic accidents caused by healthy older drivers, which consists of controlling LA for the successful management of human factors in the brain.

We calculated DSP scores at six locations on a closed-circuit track as previously described^23^. Reduced DSP scores when turning right at an intersection were only associated with parietal and occipital LA. In Japan, where left-side driving is legally enforced, more attention toward moving objects, such as vehicles driving straight in the opposite lane and pedestrians crossing the street, is required for turning right at an intersection than for other manoeuvres. When turning right, drivers with LA should drive more carefully than those without LA. In Japan, a DSP assessment with actual vehicles is legally required for the renewal of driving licences for drivers over 70 years old. The nationwide policy needs to be simplified and streamlined because the population of elderly drivers is growing rapidly^41^. Our evidence suggested that focusing on turning right at an intersection for DSP assessment may contribute to the efficient evaluation of older drivers renewing their licences.

We previously reported that healthy middle-aged adults with LA had lower scores in both tracking and sudden motion tasks of the DVC test than those without LA^20^. As shown in Fig. 3, path analyses revealed that occipital LA and BA were significantly correlated with a decline in the sudden motion task of the DVC, but not the tracking motion task, and that the sudden motion task results were significantly correlated with declines in DSP scores when turning right at intersections. The decline in visual information processing, possibly caused by occipital LA and BA, may provide further evidence that older drivers have reduced information processing speed, which might be linked to the decrease in both DVC scores in the sudden motion task and DSP scores when turning right.

The current study had several limitations. First, participants with cardiovascular diseases were excluded based on medical history data. Physiological parameters, such as electrocardiogram data, would be helpful to increase the rigor of including criteria. Second, although we examined driving performance in a real-world situation, DSP scores on a closed-circuit course while being assessed by an instructor do not completely reflect driving performances on actual roads. The present results should be validated using data reflecting the usual driving performance in private cars on commonly-used roads. Third, the sample size may not have been sufficiently large to examine the diversity of driving behaviours. However, the enrolment of 101 participants was estimated to be sufficient for analyses of the relationship between MRI data and DSP scores using actual motor vehicles, as compared with previous reports by Toyota Central Research and Keio University teams examining 39 and 32 participants, respectively^30,41^. Fourth, this study focused on older drivers. LA is most frequently diagnosed in older adults and not in young adults less than 40 years old^12,38^. However, our previous study showed that LA was significantly correlated with declining DSP scores regardless of age^23^. Therefore, all adult age groups should be investigated in further validation studies. We plan to conduct large-scale experiments with younger individuals in the near future. Finally, LA measurement uses structural data of the brain. fMRI studies may elucidate the relationship between LA and neuronal network dysfunction because damaged vessels in regions with LA induce an insufficient blood supply in the white matter, including neuronal fibres^42^. It is well known that LA coexists with vascular dementia and is considered a cause of cognitive impairment^11,12^. Comprehensive approaches using both structural and functional MRI data may further our understanding of the effects of LA on DSP.

**Figure 4:**
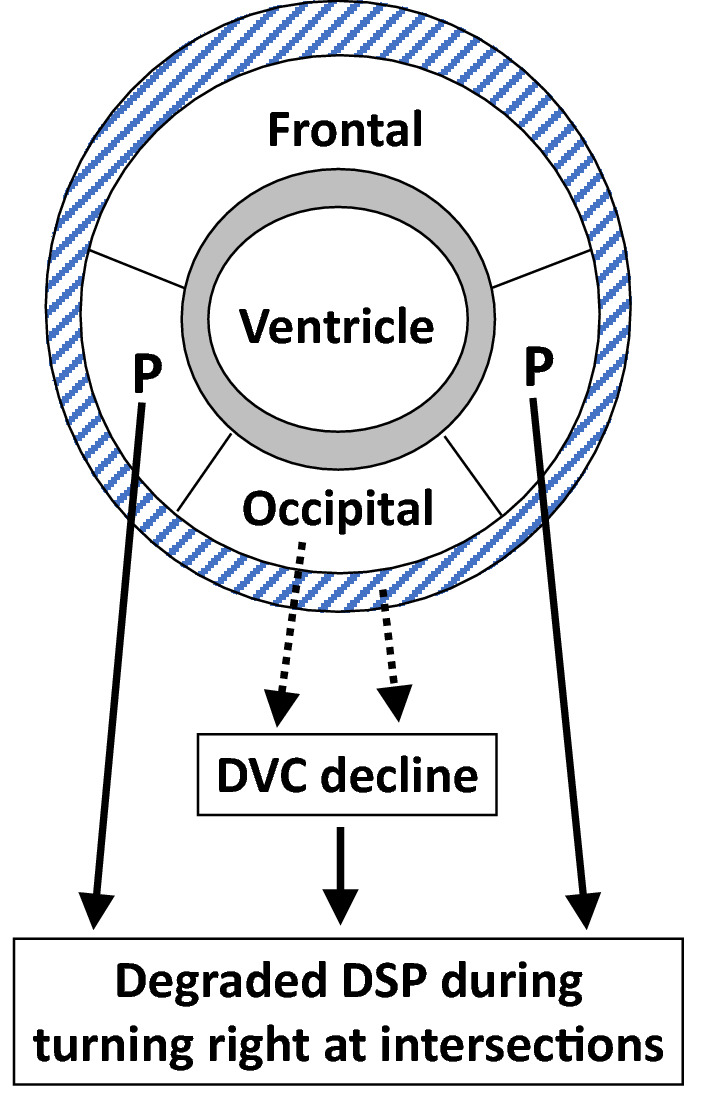
Leukoaraiosis (LA) in the parietal and occipital lobes and brain atrophy (BA) were significantly correlated with driving safety performance (DSP) and dynamic visual cognition (DVC). P, parietal lobe; Shaded zone, BA; Grey zone, periventricular LA; dashed lines, significant correlations with DVC; solid lines, significant correlations with DSP.

In conclusion, the study findings demonstrated the impactof LA, rather than ageing, for DSP. Parietal and occipital (but not frontal or temporal) LA were significantly correlated with lower DSP scores in older drivers when turning right at an intersection, although occipital LA was involved via the decline in dynamic visual cognitive function (Fig. 4). Driving a car is an essential activity of daily life for many older adults, especially among those who have difficulty walking or who live in places with poor public transportation services. The early detection of LA in the parietal and occipital lobes using MRI and the subsequent treatment of LA may be important for maintaining DSP in older adults.

## Supplementary Information

Supplementary Figure 1.

## Data Availability

The data supporting the findings of this study are available upon request from the corresponding author.
