# Supplementary material for: Parietal and occipital leukoaraiosis due to cerebral ischaemic lesions decrease the driving safety performance of healthy older adults
Source: Sci Rep. 2022 Dec 12;12:21436. doi: 10.1038/s41598-022-25899-4 (PMC9744831; doi:10.1038/s41598-022-25899-4)
Supplement: Supplementary file 1 — Supplementary Figure 1. [file 41598_2022_25899_MOESM1_ESM.pptx]

## Slide 1
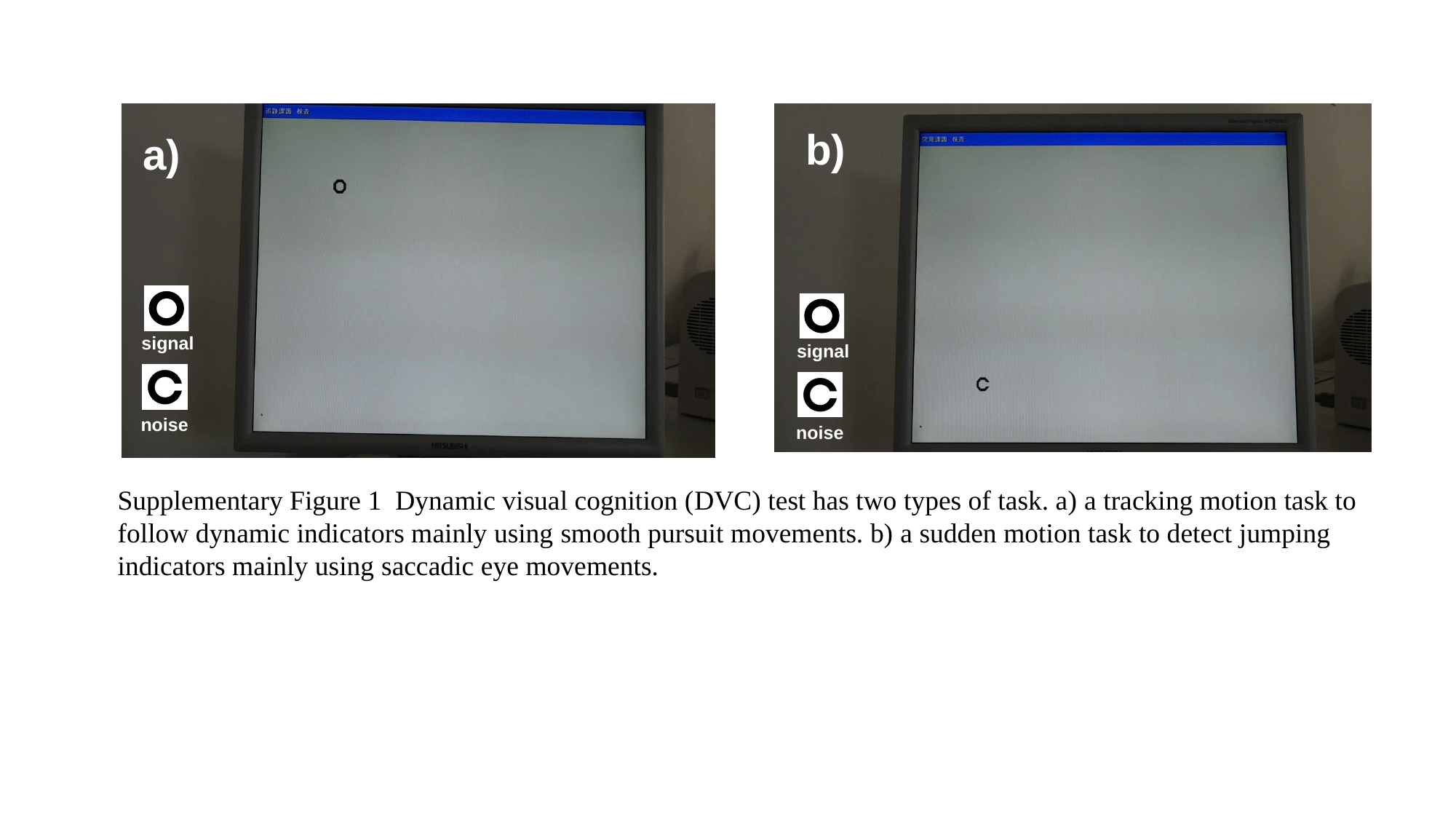

b)
a)
signal
signal
noise
noise
Supplementary Figure 1 Dynamic visual cognition (DVC) test has two types of task. a) a tracking motion task to
follow dynamic indicators mainly using smooth pursuit movements. b) a sudden motion task to detect jumping
indicators mainly using saccadic eye movements.
